# Retrospective study of tongue gangrene in Egyptian buffaloes and its surgical management

**DOI:** 10.1186/s13620-026-00336-4

**Published:** 2026-03-07

**Authors:** Marwa Abass, Reham Karam, Walaa F. Awadin, Reham Fahmy, Esam Mosbah

**Affiliations:** 1 Department of Surgery, Anesthesiology, and Radiology, Mansoura University, Mansoura, 35516 Egypt; 2 Department of Virology, Faculty of Veterinary Medicine, Mansoura University, Mansoura, 35516 Egypt; 3 Department of Pathology, Faculty of Veterinary Medicine, Mansoura University, Mansoura, 35516 Egypt; 4 Veterinary Surgery, Oncology Centre, Mansoura University, Mansoura, 35516 Egypt

**Keywords:** Lingual gangrene, Partial glossectomy, FMD, Bacteriological culture, Mycological culture

## Abstract

**Background:**

Tongue gangrene is a rare but serious condition in buffaloes. Despite its clinical and economic importance, it remains poorly documented in Egypt. This retrospective study evaluated the clinical presentation, etiological factors, histopathological findings, and surgical outcomes of tongue gangrene in Egyptian buffaloes.

**Methods:**

Forty-four buffaloes examined between January 2022 and November 2025 were classified based on the extent of tongue loss, as determined by the length (cm) of the excised gangrenous segment. Microbiological analyses included culture of deep tissue samples from the demarcation zone and rice straw samples taken from the batches fed to affected buffaloes on their farms of origin. Viral RNA was extracted from affected tongue tissues, and FMDV detection and serotyping were performed using RT-PCR targeting the 5′UTR and VP1 regions. Sequenced PCR products were analyzed using BLAST and phylogenetic reconstruction. Partial glossectomy was performed in all cases, and recovery was monitored for up to 24 weeks based on medical records.

**Results:**

The buffaloes showed systemic illness with fever and dehydration, along with characteristic dry tongue gangrene (discoloration, foul odor, loss of sensation, and a clear demarcation line). Most cases (31/44) had a recent history suggestive of FMD; the remaining cases were classified as suspected traumatic tongue injury (9/44) or no identified predisposing factor (4/44). Deep tongue tissue cultures yielded bacterial growth in 24/44 and fungal growth in 3/44 cases (presumptive identification), while rice straw samples yielded Fusarium-like fungi (12/44) and clostridia-like organisms (28/44). Histopathology confirmed coagulative necrosis with vascular congestion, bacterial colonization, and neutrophilic infiltration. FMDV serotype O was detected, showing ~ 98% identity by BLAST and clustering within the ME-SA topotype near Egy/Qalyubia/2021. Recovery time correlated significantly with the extent of tongue loss (*p* < 0.05): the shortest recovery was observed after ~ 5 cm loss, intermediate recovery after ~ 8 cm loss, and the longest recovery among survivors after ~ 10 cm loss.

**Conclusion:**

Tongue gangrene in the studied Egyptian buffaloes was most likely associated with recent FMD infection and/or traumatic tongue injuries, which may predispose to microlesions and subsequent microbial invasion. Clostridia-like organisms and Fusarium-like fungi were cultured from deep tongue tissue and from rice straw offered to the affected animals. Early diagnosis and prompt surgical intervention remain essential for successful outcomes and improved survival.

**Graphical Abstract:**

**Figure:**
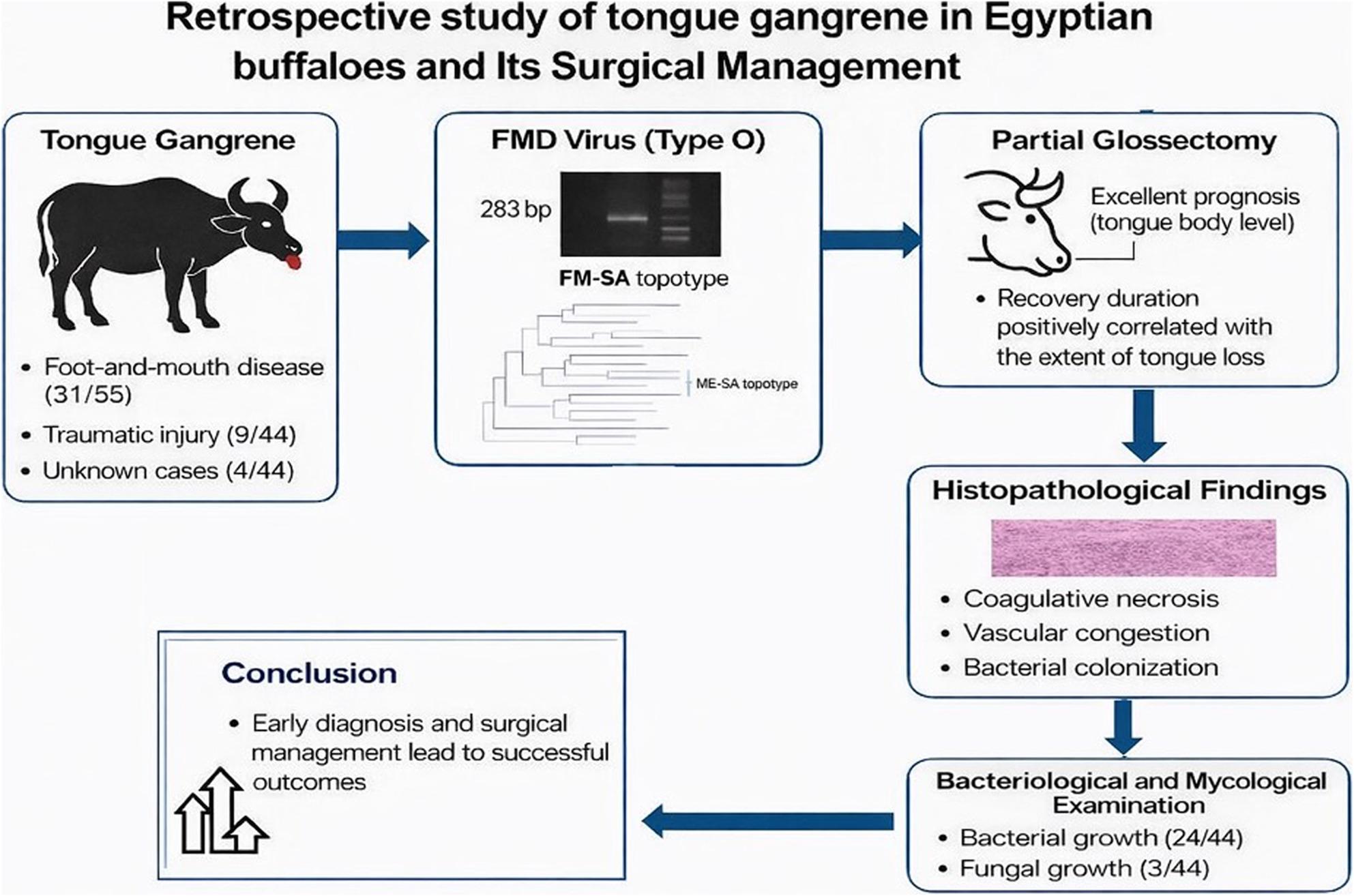
Summary of tongue gangrene in buffaloes, showing likely causes, key diagnostic findings, and outcomes of partial glossectomy

## Introduction

The Egyptian water buffalo (*Bubalus bubalis*) is a critical farm and draught animal due to its productive and reproductive roles, providing horns, hides, and skin as well as substantial quantities of meat and milk [1]. According to FAOSTAT data, the buffalo population in Egypt was approximately 3.45 million head in 2018. Although tongue gangrene is rare, it can result in serious clinical and economic consequences, including reduced feed intake, decreased milk yield, increased treatment costs, and possible culling in advanced cases [2]. The tongue is a vital and highly vascularized organ; thus, tongue gangrene typically reflects severe local injury and/or vascular compromise and may be associated with systemic disease [3–5]. Therefore, disease prevention and timely management are essential to reduce losses and protect smallholder livelihoods [6]. In buffaloes, tongue gangrene has been reported as a complication of severe oral disease, particularly neglected or poorly managed Foot and mouth disease (FMD), and it may also occur in gangrenous syndromes linked to ingestion of moldy rice/paddy straw (Degnala disease) [7–9]. Secondary bacterial contamination can aggravate necrosis and worsen the clinical course [9, 10].

FMD is an obstacle to worldwide trade and affects productivity and general condition in cloven-hoofed animals [11]. It is rarely fatal in adults but may cause mortality in young animals [11].

Clinical signs commonly include vesicular lesions in the oral cavity and feet, fever, lameness, and a drop in milk production [12]. FMDassociated circulatory disturbances and tissue edema have been described and may contribute to tissue compromise in severe cases, potentially facilitating progression of lingual lesions [13, 14]. Because serotype distribution varies across outbreaks and regions, incorporating locally circulating serotypes, particularly O, A, and SAT2 in Egypt, into molecular typing panels is essential for accurate diagnosis and informed control measures [15, 16].

Degnala disease (gangrenous syndrome) is a fungal-associated condition (mycotoxicosis) linked to ingestion of moldy/contaminated paddy straw, and buffaloes may show more severe clinical manifestations [9, 10]. The clinical course is variable: lesions often improve within weeks after withdrawal of contaminated straw and supportive management, whereas severe cases may be prolonged, with lesions occasionally persisting for up to 32 months, and may be fatal [10, 17].

Typical lesions involve lameness, edema, and gangrenous ulceration of distal parts; in some cases, the muzzle and tip of the tongue may be involved [9, 17]. Because severe FMD-associated oral lesions and Degnala-related gangrenous syndromes can overlap clinically -particularly when necrosis and secondary bacterial contamination are present- targeted clinicopathological and microbiological characterization is important for early diagnosis and appropriate intervention [10, 17]. Despite the clinical and economic importance of tongue gangrene in buffaloes, the condition remains poorly documented in Egypt. Most existing reports focus on general FMD outbreaks or Degnala disease, with limited information regarding the specific clinical presentation, pathological features, microbiological findings, and surgical outcomes of tongue gangrene in buffaloes in Egypt [18, 19].

Moreover, retrospective analyses evaluating prognostic indicators and recovery patterns following partial glossectomy are lacking. These gaps highlight the need for a comprehensive description of this condition to support early diagnosis and improve treatment decisions. Therefore, the present study aimed to retrospectively evaluate the clinical, pathological, and microbiological characteristics of tongue gangrene in Egyptian buffaloes and to assess the effectiveness of surgical management and post-operative recovery outcomes.

## Materials and methods

### Ethical approval

All procedures in this study have been approved by the Mansoura University Animal Care and Use Committee with a documented code MU-ACUC (VM.R.25.11.253). All methods were carried out in accordance with relevant guidelines and regulations. All methods are reported in accordance with ARRIVE guidelines. This work represents a retrospective analysis of clinical cases and medical records. At admission, owners provided written routine clinical/surgical consent for examination, treatment, and the indicated surgical intervention, and they also provided written permission for the use of clinical images and anonymized data for scientific purposes (research/publication). Subsequently, data were extracted from the records and analyzed retrospectively in a de-identified manner.

### Animals

In this retrospective study of forty-four female buffaloes from the Dakahlia governorate, Egypt, presented to the Shoha Veterinary Hospital between Jan 22 and Nov 25, were included.

### Inclusion and exclusion criteria

Buffaloes with clinically confirmed dry gangrene of the tongue, complete medical records, and documented surgical intervention were included in this study. Animals were excluded if their clinical files were incomplete; if tongue lesions were not clearly diagnostic for gangrene; if more than half of the tongue was affected, or if they suffered from multiple erosions in different body parts (hoof, tail, skin).

### Perioperative preparation

All included cases underwent partial glossectomy (resection of the gangrenous portion) as the standard surgical management for dry tongue gangrene. Before surgery, physiological parameters such as heart rate (HR, beats/min), respiratory rate (RR, breaths/min), rectal temperature (RT, °C), skin dehydration test (SDT, s), and capillary refill time (CRT, s) were recorded.

Intravenous fluids were administered over 4–6 h at a total volume of 20–40 mL/kg, based on the estimated degree of dehydration [20]. The total volume was divided equally between 0.9% NaCl and Ringer’s lactate and given intravenously one after the other. Preoperatively, buffaloes received a single intramuscular dose of amoxicillin sodium 15% (Vetrimoxin LA; CEVA Animal Health, France) at 15 mg/kg. Postoperatively, for 5 consecutive days, buffaloes received IM flunixin meglumine (Flunixin; Norbrook^®^ Laboratories Limited, United Kingdom) at 2 mg/kg and oxytetracycline 20% (Oxytetra LA; Kela Laboratoria, Belgium) at 20 mg/kg [21, 22].

### Surgical intervention (partial glossectomy)

**Fig. 1:**
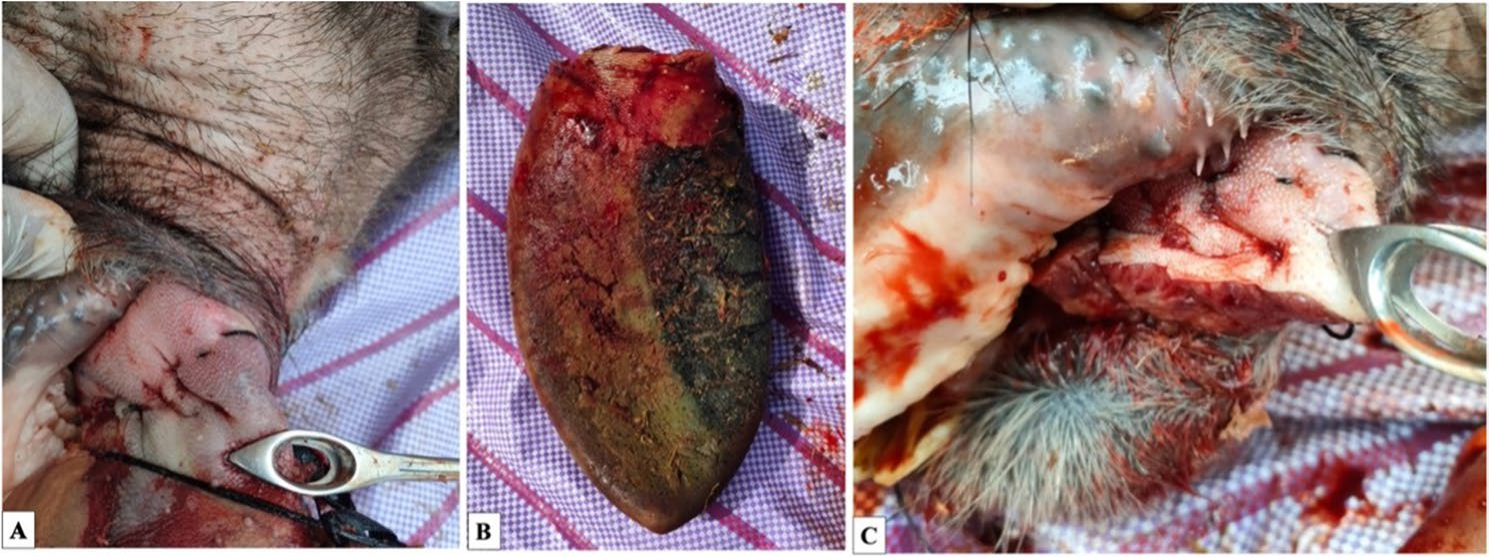
Partial glossectomy of the gangrenous portion of the tongue in sedated buffaloes in right lateral recumbency **(A–C)**.** A** A simple continuous suture placed proximal to the demarcation line acts as a temporary tourniquet. **B** The tongue after excision of the gangrenous portion. **C** Two-layer closure of the remaining healthy tongue using interrupted horizontal mattress and simple continuous suture patterns

Each buffalo was sedated with an IM injection of xylazine HCl 2% (Xyla-Ject, Adwia Company, Egypt) at a dose of 0.05 mg/kg, then positioned in right lateral recumbency, and the head and limbs were secured with ropes. The oral cavity was opened using a mouth gag. The tongue was grasped, and a simple continuous suture was placed proximal to the demarcation line to act as a temporary tourniquet. Lavage of the tongue was repeated several times perioperatively using a solution of 500 mL physiological saline (0.9% NaCl; Egypt Otsuka Pharmaceutical Co., Egypt) mixed with 55 mL Povidone-iodine 10% (Betadine; Amoun Co., Egypt). A circumferential (ring block) submucosal infiltration was performed using 10 mL lidocaine HCl 2% (Debocaine; Sigma-Tec Pharmaceutical Indust. Co., Egypt) injected on the right and left aspects of the tongue at the planned transection level, rostral to the tourniquet and caudal to the demarcation line. Partial glossectomy was performed by surgical amputation of the gangrenous portion of the tongue. The surgical site was closed in two layers using Polyglycolic acid USP 2 (Egysorb; Taiser-Med SAE) on a round-bodied needle, with the first layer in an interrupted horizontal mattress pattern and the second layer in a simple continuous pattern. After removal of the tourniquet, the tongue was gently massaged to restore circulation and promote drainage of blood pooled rostral to the tourniquet during its application [23]. Postoperatively, animals were kept on soft green fodder for 7 days to protect the wound and reduce mechanical trauma/contamination from coarse feed (Fig. 1).

### Outcome measures

Follow-up information was retrieved retrospectively from medical records. Animals were re-examined at approximately days 7 and 14 postoperatively and thereafter at monthly intervals, with follow-up data available for up to 24 weeks. Follow-up duration varied depending on case availability. Recorded parameters included (i) wound healing status; (ii) surgery-related postoperative complications; (iii) feeding ability; (iv) body weight and general condition; (v) survival/return to the herd; and (vi) recovery time. These were defined as follows: (i) wound healing status (normal vs. delayed healing). Delayed healing was defined as persistence of inflammation, infection, or dehiscence as recorded at scheduled recheck visits; (ii) surgery-related postoperative complications (hemorrhage, wound dehiscence, infection, and excessive salivation); (iii) feeding ability (return to normal prehension and mastication and absence of dysphagia). Feeding ability was assessed clinically based on the ability to prehend and masticate normally and swallow without difficulty as documented in the medical records; (iv) body weight and general condition; (v) survival/return to the herd; and (vi) recovery time (weeks), defined as the interval from surgery to restoration of functional feeding with improvement in body condition. In addition, tongue tissue samples were subjected to bacteriological and mycological culture and virological molecular detection and typing, as described below.

### Grouping of cases

Animals were categorized into four groups according to the extent of tongue loss (length of the excised portion, cm). Measurements were taken from the tongue apex to the demarcation line. Group 1 (first part of the tongue apex; 5 ± 1.4 cm, *n* = 27), Group 2 (middle portion of the apex; 8 ± 1.1 cm, *n* = 9), Group 3 (tongue body; 10 ± 0.7 cm, *n* = 5), and Group 4 (up to the frenulum linguae; 12 ± 1.2 cm, *n* = 3). These groups were used for comparison of recovery time.

### Histopathological examination

Gangrenous tongue samples were fixed in 10% neutral buffered formalin for histopathological examination. Formalin-fixed tissues were routinely processed by dehydration through ascending grades of ethanol, cleared in xylene, embedded in paraffin wax, sectioned at 4–5 μm, mounted on glass slides, and stained with hematoxylin and eosin (H&E). The sections were examined under a light microscope, and lesions were recorded [24].

### Bacteriological and mycological examination

Deep tissue samples for bacteriological and mycological examinations were collected aseptically from the demarcation zone (junction between viable and necrotic tongue tissue) in all cases (*n* = 44) using small tissue pieces, after gentle surface cleansing with sterile normal saline to minimize contamination. This site was selected to maximize recovery of the etiologic agents and reduce contamination from the superficial necrotic surface. Samples were placed in sterile tubes, transported on ice, and processed within 2–4 h of collection. For bacteriological examination, samples were cultured aerobically at 37 °C on standard nutrient agar, MacConkey agar, and blood agar (Thermo Fisher Scientific, USA). In addition, anaerobic culture was performed on Reinforced Clostridial Medium (RCM) agar and incubated anaerobically at 37 °C. Anaerobiosis was achieved using an anaerobic jar with gas-generating sachets. Identification was presumptive based on colony morphology (culture characteristics) and microscopic examination, Gram staining; no biochemical/molecular confirmation was performed. For mycological examination, samples were cultured on Sabouraud Dextrose Agar (SDA) and SDA supplemented with cycloheximide and chloramphenicol (SDACC) and incubated at 27 °C and 37 °C in a biological oxygen demand (BOD) incubator [25, 26].

Additionally, rice straw samples (*n* = 44) were collected from affected premises and processed within 2–4 h. For mycology, samples were cultured on SDA and SDACC at 27 ± 2 °C. Microscopic identification was performed using lactophenol cotton blue mounts, and conidial dimensions were measured to support presumptive identification of Fusarium spp. For anaerobic bacteriology, samples were cultured on RCM agar and incubated anaerobically at 37 °C under the same anaerobic conditions described above. Identification was based on colony morphology and microscopic examination/Gram staining, without biochemical/molecular confirmation [25, 26].

### Viral RNA extraction

Tongue tissues collected were homogenized and clarified according to the recommended protocol, where 200 µL supernatant was collected. Aliquots of the clarified homogenates were stored at − 80 °C until further molecular analysis (FMDV RT-PCR and VP1 sequencing). QIAamp^®^ Viral RNA Mini Kit (Qiagen, Valencia, USA, lot no. 145026969) was used for viral RNA extraction according to manufacturer instructions. The eluted 50 µL RNA was used for further virus detection and typing.

### Detection of FMDV and typing

Partial amplification of the viral 5` UTR and partial VP1 was done for viral detection and typing, respectively. Topscript one-step RT-PCR mastermix (Enzynomics, South Korea, Cat No. RT4105) was used for this purpose to generate 20 µL reactions. Primers used for the detection of the virus or typing are listed in Table 1.

**Table 1:** Primers used in the molecular typing and identification of the excised sample:

Target	Primer	Sequence	Product	Reference
Detection:5′UTR	F1	5’-GCCTGGTCTTTCCAGGTCT-3’	328 bp	[27]
Detection (5′UTR)	R1	5’-CCA GTC CCC TTC TCA GAT C-3		
Typing: O	O-EA-F	5’-CCTCCTTCAAYTACGGTG-3’	283 bp	[28]
Typing A	A-Egy-F	GGAATCWGCAGACCCTGTC	750 bp	[29]
Typing SAT-2	SAT2-Egy-F	TGAYCGCAGTACACAYGTYC	666 bp	[29]
Reverse of O, A, SAT-2	NK61	5’-GACATGTCCTCCTGCATCTG-3’		

The thermal profile adopted in PCR reactions was reverse transcription at 50 °C for 30 min, initial denaturation at 95 °C for 15 min, then a PCR cycle of denaturation at 95 °C for 30 s, annealing at 60 °C for all primer pairs for 30 s, then an extension step at 72 °C for 30 s. A final extension at 65 °C for 5 min was performed according to the kit protocol. Positive control type O FMDV was supplied by the virology department, Animal Health Research Institute, Dokki, Giza, to be added to the detection and typing PCR reactions. A no-template control (NTC) was included in each run.

The amplified products were allowed to migrate in a 1.5% agarose gel stained with EtBr in an electric field supplied with 1x TBE buffer for 50 min. After migration, amplified products were visualized under a UV transilluminator.

### PCR product purification

QiaQuick PCR product purification kit (Qiagen Inc., Valencia, CA) was used to purify amplified products’ DNA according to kit recommendations.

### Sequencing reaction and phylogenetic analysis

Purified PCR amplicons were sequenced in both forward and reverse directions using an Applied Biosystems 3500 automated DNA sequencer (ABI 3500, USA) and the BigDye Terminator v3.1 Cycle Sequencing Kit (Applied Biosystems, Foster City, CA; Cat. No. 4336817), according to the manufacturer’s instructions. Sequencing reactions were performed using the corresponding PCR primers (Table 1).

Raw chromatogram files (AB1) were visually inspected for quality, and low-quality ends were trimmed before downstream analysis. Forward and reverse reads were assembled into a consensus sequence, and ambiguous base calls were resolved. Consensus sequences were compared with GenBank entries using BLASTn (Basic Local Alignment Search Tool) to determine the closest sequence identity [30]. For phylogenetic analysis, sequences were aligned in MEGA 11, and a phylogenetic tree was constructed using the Neighbor-Joining method with 1,000 bootstrap replicates to evaluate node support [31].

### Statistical analysis

Data analysis was performed using SPSS software version 26. Data distribution was assessed for normality using the Shapiro–Wilk test. Normally distributed quantitative variables (e.g., tongue-loss “cm”, age, and recovery duration) were expressed as mean ± SD, whereas non-normally distributed variables were expressed as median (IQR). Vital signs (rectal temperature, heart rate, and respiratory rate) were reported as median (IQR; range). Recovery time was compared among the four tongue-loss groups using one-way ANOVA (Tukey’s post-hoc test). If data were not normally distributed, the Kruskal–Wallis test (with appropriate post-hoc comparisons) was used. Correlation between tongue loss and recovery duration was assessed using Pearson’s correlation coefficient for normally distributed data; otherwise, Spearman’s rank correlation was used. A p-value < 0.05 was considered statistically significant. Group 4 was excluded from recovery-time analysis due to unavailable follow-up data (slaughter before recovery assessment).

## Results

**Fig. 2:**
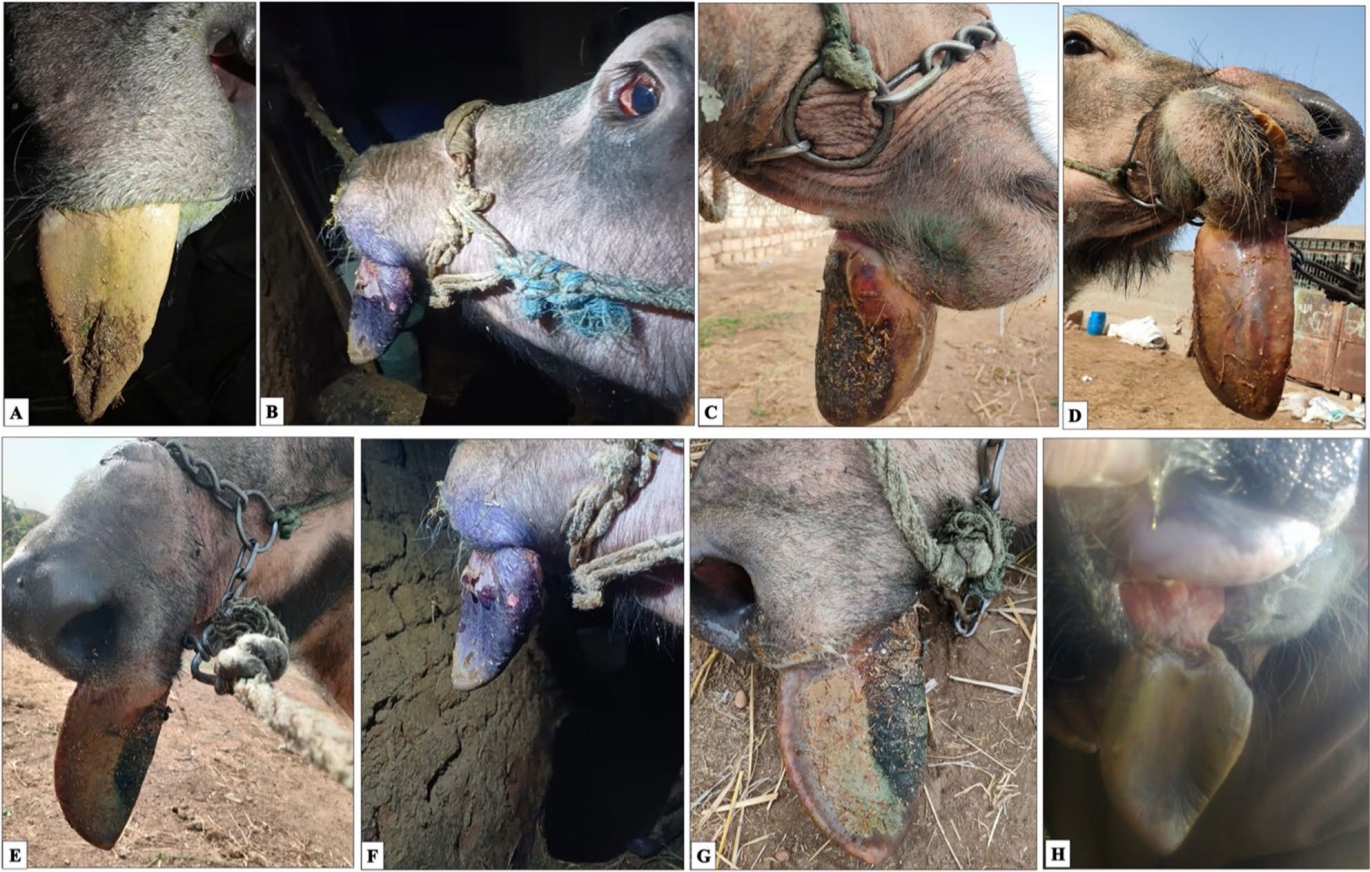
Gangrenous tongue in buffaloes showing different presentations. The tongue is covered with necrotic debris **(A–H)**. Protruded tongue showing first portion of the tongue affected **(A);** Tongue with gangrene up to the middle with congested conjunctiva **(B);** Complete protrusion of tongue apex **(C);** Protrusion extending to the tongue body and frenulum linguae (**D**). Ulcerated wounds on the tongue surface (**E** & **F**). Dark color with a clear line of demarcation (**G**); Partial sloughing of the necrotic portion **(H)**

The animals enrolled as cases in this study exhibited dullness, loss of appetite, decreased milk production, stiffness, lameness, emaciation, fever, and a rough coat. Additionally, parts of their tongues were protruding, enlarged, dark-colored, cold, emitting an offensive odor, lacking sensation, and had a distinct line of demarcation (Fig. 2).

Predisposing factors were identified in most cases. Thirty-one (31) buffaloes had a history of vesicular lesions with cracks on their hooves due to FMD a month earlier. The remaining 13 buffaloes had no owner-reported history consistent with FMD; nine (9) buffaloes had a history of tongue injuries attributed to mechanical trauma from rice straw that was suspected to be contaminated (laboratory findings were consistent with fungal contamination), or other sharp objects such as nails. These findings indicate contamination but do not establish causality. In four (4) buffaloes, no apparent predisposing factors were identified, and the underlying etiology remained undetermined.

According to owners, the condition started with mild oral ulcers and anorexia, then progressed to tongue protrusion, and the clinic was contacted approximately 9–18 days after onset because of poor response to self-administered empirical treatment that varied between cases. Reported treatment including intramuscular antibiotics for 3 days (e.g., procaine penicillin G or ceftiofur sodium) and intramuscular anti-inflammatory drugs for 3 days (e.g., meloxicam or ketoprofen). In addition, owners applied a topical antimicrobial spray and performed oral rinsing using a 2% sodium bicarbonate solution and gentian violet; some owners also added lemon.

Vital signs were recorded at admission using a standardized protocol, and most cases presented at a similar stage. Clinical examination (*n* = 44) revealed vital signs as follows: median rectal temperature 40.5 °C (median; IQR: 39.9–40.7; range: 39.8–41.0), median heart rate 91 (IQR: 89–94; range: 88–95) beats/min, and median respiratory rate 35 (IQR: 33–36; range: 33–37) breaths/min. In all animals, the CRT was less than 1 s, while the SDT exceeded 7 s, indicating mild dehydration (approximately 5–6%) and the mucous membranes appeared semidry and congested.

Tongue protrusion varied among animals, reaching different anatomical levels. Twenty-seven (61.3%) buffaloes showed a protrusion of the tip of the apex of the tongue, measuring 5 ± 1.4 cm in length. Nine (20.5%) buffaloes had a protruded tongue reaching the middle portion of the apex, measuring 8 ± 1.1 cm in length. Five (11.4%) buffaloes exhibited a protruded tongue reaching the body of the tongue, measuring 10 ± 0.7 cm in length. Three (6.8%) buffaloes showed a protruded tongue extending up to the frenulum linguae with an average length of 12 ± 1.2 cm.

**Fig. 3:**
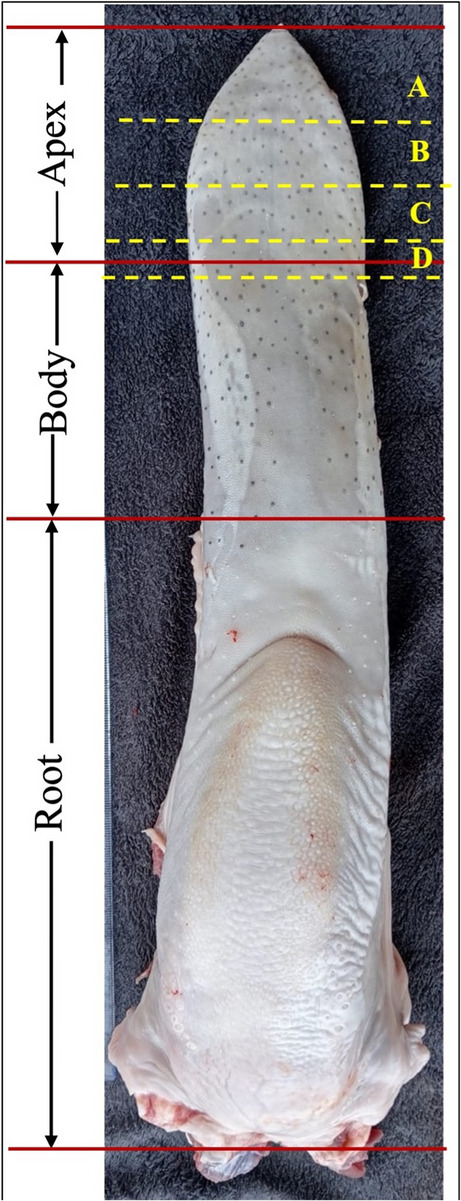
A schematic diagram illustrating the main parts of the tongue (apex, body, root). The level of partial glossectomy (A= removal of the apical tip, B= removal of the middle portion of the apex, C= removal of the entire apex, D= removal of the apex extending up to the frenulum linguae

All cases had dry gangrene; consequently, the partial glossectomy was recommended depending on the line of demarcation (Fig. 3).

During the 24-week follow-up period, wound healing was uneventful, and no surgery-related postoperative complications (hemorrhage, wound dehiscence, infection, excessive salivation, or dysphagia) were recorded. The main adverse outcome was functional feeding impairment associated with greater tongue loss (≥ 12 cm); three buffaloes (3/44; 6.8%) failed to regain body weight and general condition due to impaired feeding ability, and animals were slaughtered after stabilization of their general condition (Table 2).

**Table 2:** Clinical classification and outcome of 44 buffaloes affected by gangrenous tongue

Items	Forty-four buffaloes affected with gangrenous tongue			
	Group 1	Group 2	Group 3	Group 4
No. of case (%)	*N* = 27 (61.3%)	*N* = 9 (20.5%)	*N* = 5 (11.4%)	*N* = 3 (6.8%)
Age (year)	6 ± 0.4	5 ± 1.2	8.5 ± 0.3	7.2 ± 0.8
Length of tongue loss (cm)	5 ± 1.4 cm	8 ± 1.1 cm	10 ± 0.7 cm	12 ± 1.2 cm
Case history	▪ Sharp object injuries (*N* = 5) ▪ FMD ulcer (*N* = 22)	▪ Sharp object injuries (*N* = 2) ▪ FMD ulcer (*N* = 7)	▪ Sharp object injuries (*N* = 2) ▪ FMD ulcer (*N* = 2) ▪ Unknown cause (*N* = 1)	▪ Unknown cause (*N* = 3).
Postoperative outcome (feeding ability/ body condition)	▪ Animals can regain body weight. ▪ Normal feeding restored.	▪ Animals regained body weight. ▪ Fed mainly on soft, digestible concentrate ▪ Partial feeding ability.	▪ Fed on soft, easily digestible concentrate. ▪ Impairment of feeding ability.	▪ Unable to prehend or chew normally ▪ Animals were unable to regain body weight. ▪ Animals were slaughtered.
Recovery time (week)	6 ± 2.0 weeks	10 ± 1.0 weeks	18 ± 2.5 weeks	Not assessed (slaughtered)
Prognosis	Excellent	Good	Fair	Poor

Recovery time differed significantly among tongue-loss groups in surviving animals (one-way ANOVA, *p* < 0.05; Tukey’s post-hoc test). Animals with smaller tongue loss recovered faster: Group 1 (5 ± 1.4 cm) recovered in 6 ± 2.0 weeks, Group 2 (8 ± 1.1 cm) in 10 ± 1.0 weeks, and Group 3 (10 ± 0.7 cm) in 18 ± 2.5 weeks. Tukey’s post-hoc test confirmed significant differences between groups 1–3. Recovery duration was positively correlated with tongue loss (excised length) (*r* = 0.73, *p* < 0.001), indicating that greater tongue loss was associated with a longer recovery period. Group 4 animals (12 ± 1.2 cm; *n* = 3) were slaughtered; therefore, they were excluded from recovery-time analysis.

- Values are expressed as mean ± SD. FMD: Foot-and-mouth disease. Loss of tongue was measured from the apex to the necrotic margin. Follow-up period: 24 weeks.
- Recovery time decreased as tongue loss decreased; accordingly, tongue loss (excised length) was positively correlated with recovery duration (*r* = 0.73, *p* < 0.001).
- All outcome variables were assessed postoperatively during the 24-week follow-up period.

### Histopathological examination

**Fig. 4:**
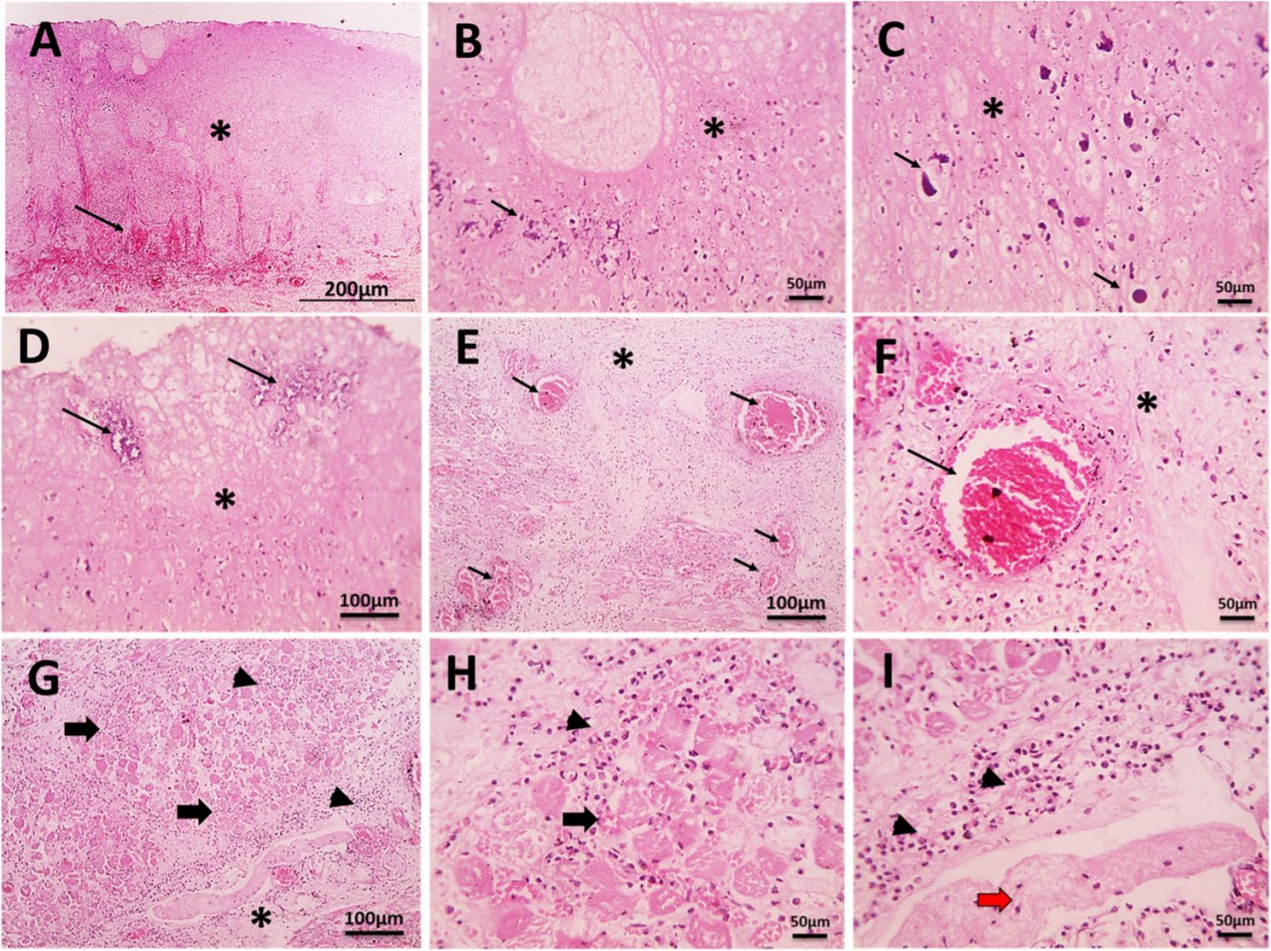
Microscopic pictures of **H**&**E**-stained tongue sections. **A**: 40x, **E** & **G**: 100x, and **B**, **C**, **D**, **F**, **H**, & **I**: 400x. **A**: coagulative necrosis of the stratified squamous epithelium (*) with underlying congested vascular vessels. **B**: nuclear debris (arrow) in the area of necrosis (*). **C**: foci of calcification (arrow). **D**: bacterial colonization (arrow) in the area of necrosis. E: interstitial congestion (arrows), edema (*). **F**: Fibrin thrombus within a congested blood vessel (arrow) surrounded by necrotic tissue. **G** & **H**: Coagulative necrosis of muscle fibers (arrows) surrounded by edema (*) and neutrophilic infiltration (arrowheads).** I**: fibrin thrombus (arrow) surrounded by edema (*) and neutrophilic infiltration (arrowheads)

Microscopically, the tongue sections revealed coagulative necrosis of the covering epithelium with nuclear debris, foci of calcification, and bacterial colonization. Coagulative necrosis of muscle fibers was seen with marked interstitial congestion, edema, hemorrhage, fibrin thrombi, and neutrophilic infiltration (Fig. 4A-I).

### Bacteriological and mycological findings

Deep tongue tissue samples collected from the demarcation zone (*n* = 44) yielded bacterial growth in 24/44 cases (54.5%). On anaerobic culture (RCM), the growth was characterized as mixed growth with large Gram-positive rods suggestive of clostridia (Clostridium-like organisms) based on colony morphology and Gram staining. Fungal growth was recovered from 3/44 cases (6.8%) on Sabouraud Dextrose Agar (SDA) and Sabouraud Dextrose Agar supplemented with chloramphenicol and cycloheximide (SDACC). Presumptive identification was based on culture characteristics and microscopic examination. Aerobic cultures yielded mixed bacterial growth without definitive identification.

Rice straw samples (*n* = 44) collected from premises housing affected buffaloes yielded characteristic Fusarium-like colonies in 12/44 samples (27.3%) within 48 h of incubation on SDA at 27 ± 2 °C. In contrast, colony development on SDACC only appeared after 96 h, likely reflecting inhibitory effects of cycloheximide-containing selective media. Microscopic examination of lactophenol cotton blue–stained preparations revealed microconidia (9.89 ± 0.89 μm) and macroconidia (21.94 ± 2.66 μm) consistent with *Fusarium* spp.

In addition, rice straw samples (*n* = 44) had clostridia-like colonies in 28/44 samples (63.6%) after incubation on Reinforced Clostridial Medium (RCM) agar under anaerobic conditions at 37 °C. Typical colonies appeared within 24 to 48 h. Microscopic examination of Gram-stained smears revealed large Gram-positive rod-shaped bacteria suggestive of clostridia (Clostridium-like organisms) without confirmatory identification (Table 3).

**Table 3:** Bacteriological and mycological findings of deep tongue tissue and rice straw samples (*n* = 44)

Sample type (*N* = 44)	Target / finding	Positive (*n*/*N*)	%
Deep tongue tissue	Bacterial growth (mixed; large Gram-positive rods suggestive of clostridia [Clostridium-like organisms] on anaerobic culture RCM)	24/44	54.5
	Fungal growth on SDA/SDACC	3/44	6.8
Rice straw	Clostridia-like colonies on RCM (anaerobic)	28/44	63.6
	Fusarium-like colonies on SDA (48 h) with delayed growth on SDACC (96 h)	12/44	27.3

### Molecular detection, typing, and sequencing findings

Clinically, 31/44 buffaloes had a reported history consistent with FMD one month earlier; there was successful amplification of the partial 5`UTR indicated positive molecular detection of FMDV in the excised tissues, with a band at the expected size (328 bp). Type-specific RT-PCR yielded an amplicon of the expected size (283 bp), consistent with FMDV serotype O. BLASTn analysis of the consensus sequence showed ~ 98% nucleotide identity to FMDV serotype O sequences in GenBank [30].

Type O FMDV sequences representing different topotypes were collected from GenBank in addition to recent type O sequences from Egypt for phylogenetic analyses.

**Fig. 5:**
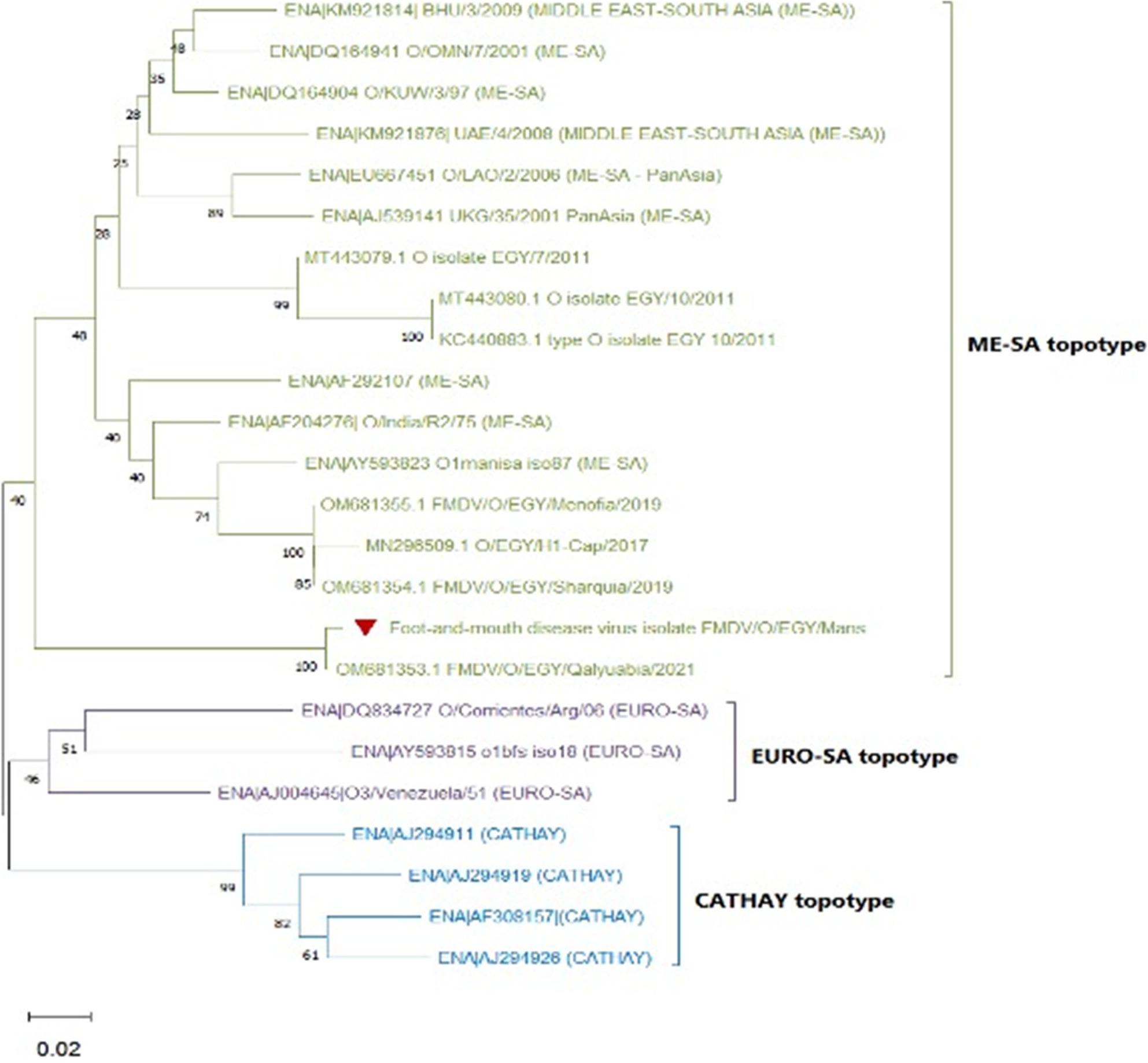
Phylogenetic tree of the identified type O FMDV

Phylogeny inferred that the obtained sequence clustered within the ME-SA (Middle East–South Asia) topotype and in the same clade as Egy/Qalyubia/2021 (Fig. 5).

## Discussion

Lingual gangrene in bovines is an uncommon but serious condition that is typically associated with severe oral lesions (notably advanced FMD) and compounded by secondary bacterial infection, with prognosis largely influenced by the extent of tissue involvement [32, 33]. The present retrospective case series documents clinically evident necrosis and dry gangrene of the tongue in buffaloes examined in Dakahlia Governorate during 2022–2025, and integrates clinical findings, histopathology, microbiology, and molecular evidence to explore the most likely contributing factors and prognostic indicators.

In this case series, most buffaloes had a history of FMD infection (70.5%), while a smaller proportion had a history of traumatic tongue wounds (22.7%), suggesting that severe post-FMD ulceration and/or mechanical injury may predispose to mucosal disruption and deeper tissue involvement [12, 34, 35]. However, no apparent predisposing factor was identified in 3/44 cases (6.8%).

Lingual gangrene following FMD has been attributed to vascular thrombosis secondary to severe ulceration, leading to local ischemia and progressive tissue necrosis [12, 13, 36]. The clinical presentation observed in this study—dark discoloration, foul odor, coldness, loss of sensation, and a distinct line of demarcation—matches the classical description of dry gangrene and is consistent with infection-associated vascular compromise leading to ischemia, with subsequent secondary microbial infection [37]. Given that a substantial proportion of cases had a recent history suggestive of FMD and that FMD is known to cause vascular and epithelial damage, it is highly likely that there is a causal association between recent FMD infection and the subsequent development of lingual gangrene in many cases. Importantly, because this was a retrospective case series without unaffected controls, a definitive causal relationship cannot be confirmed however, the findings are strongly suggestive of a causal association between recent FMD infection and subsequent lingual gangrene in many cases. Stressors such as thermal, nutritional, and metabolic challenges can impair immune responses and increase susceptibility to opportunistic infections in ruminants [38]. Opportunistic pathogens, including Fusobacterium spp., have been implicated in necrotizing glossitis in bovines [18], and comparable ulcerative lingual lesions are more frequent in animals in poor condition with delayed healing [39]. In the present study, the combination of mucosal injury (post-FMD or trauma) and immune compromise likely created favorable conditions for progression from ulceration to necrosis and gangrene, with secondary microbial colonization of devitalized or traumatized tissues contributing to lesion progression.

Histopathological examination confirmed coagulative necrosis of the epithelium and muscle fibers with bacterial colonization, vascular congestion, fibrin thrombi, and neutrophilic infiltration. This pattern supports ischemic necrosis complicated by secondary infection and aligns with microscopic findings described in bovine necrotic glossitis and gangrenous stomatitis [40]. These results strengthen the interpretation that vascular compromise is a central driver, while microbial invasion contributes to lesion expansion and systemic illness.

Secondary bacterial infection is expected in devitalized, poorly perfused tissues, where anaerobic conditions promote mixed growth. In this study, anaerobic culture yielded large Gram-positive rods consistent with clostridia-like organisms; however, species-level confirmation was not performed. These findings are therefore best interpreted as evidence of anaerobic secondary infection contributing to tissue destruction and lesion progression, rather than proof of a single primary etiological agent. Given this, systemic oxytetracycline is commonly recommended in cases of lingual gangrene/necrotic glossitis because of its activity against anaerobic bacteria implicated in secondary infection and tissue necrosis [12].

A previous report suggested that a buffalo lingual gangrenous syndrome could be linked to mycotoxins produced by Fusarium spp. isolated from rice straw [7]. However, *Fusarium* spp. and other saprophytic fungi are commonly recovered from rice straw and other roughage on many farms without overt disease; therefore, their isolation from rice straw alone cannot be considered evidence of a causal relationship with lingual gangrene [34, 35].

In the present study, Fusarium-like colonies from rice straw samples indicate fungal contamination, which may be associated with mucosal irritation and delayed healing [10]. However, this finding may be incidental and should be interpreted cautiously, given the lack of control samples from unaffected premises. Preventive strategies for buffalo lingual gangrene in rice-straw–fed systems should emphasize rice straw quality control and minimizing fungal contamination; when mycotoxin contamination is suspected, sorbents such as hydrated sodium calcium aluminosilicate (HSCAS) have been recommended to reduce the gastrointestinal impact of aflatoxins [41].

In dry gangrene, partial glossectomy and debridement are often essential to remove necrotic tissue and prevent septic complications [12, 13, 42]. In the present study, partial glossectomy performed according to the demarcation line led to variable outcomes primarily determined by the extent of tissue loss. A significant positive correlation was observed between excised tongue length and recovery duration (*r* = 0.73, *p* < 0.001; *n* = 44). Buffaloes with smaller losses regained feeding behavior within two to six weeks, whereas those with larger losses exhibited impaired prehension and mastication and failed to regain body weight. These findings agree with previous reports that the extent of tissue loss directly affects tongue mobility, saliva distribution, and the ability to prehend coarse feed [12, 43].

Clinically, all affected buffaloes showed fever, tachycardia, tachypnea, congested mucous membranes, and dehydration, consistent with systemic illness accompanying gangrenous conditions [44]. Although these findings are compatible with systemic inflammatory response and possible toxemia in severe cases, toxemia was not systematically assessed using standardized laboratory parameters (e.g., hematology, serum biochemistry, acid–base status, or inflammatory biomarkers). This is an important limitation, particularly for animals with extensive lesions (e.g., ≥ 12 cm excision length), where toxemia and septic complications would be expected to be more likely. Future work should incorporate objective systemic indices to better link lesion extent, microbial burden, and systemic compromise.

Although owner-reported onset-to-presentation intervals were available, they were not documented in a standardized manner, limiting formal analysis of symptom-to-treatment interval as a prognostic factor. Therefore, statements regarding “early intervention” should be interpreted cautiously; within this series, the extent of devitalized tissue (excised length) emerged as the most practical prognostic indicator.

Molecular detection of FMDV serotype O in excised tissues supports an association between FMD infection and lingual gangrene in a substantial proportion of cases but does not prove causation. Phylogenetic analysis clustered the isolate within the ME-SA topotype and in the same clade as the Egyptian strain Egy/Qalyubia/2021, suggesting persistent circulation of this lineage in Egypt [45, 46]. Although primers targeting multiple FMDV serotypes were used, only serotype O was identified in the examined tissues, which aligns with epidemiological reports indicating the predominance of type O (ME-SA) viruses in Egyptian livestock in recent years [37].Notably, although primers targeting multiple FMDV serotypes were used, only serotype O was detected in the examined tissues; this is consistent with reports indicating the predominance of type O (ME-SA) viruses in Egyptian livestock in recent years [37]. Failure to detect other serotypes in this series may reflect the prevailing field epidemiology during the study period and/or factors related to sampling timing and tissue viral load; therefore, the present molecular results support an association with serotype O in these cases, without excluding the potential involvement of other serotypes in different outbreaks or sampling stages.

This was a retrospective analysis of clinical cases treated under routine veterinary care; bacteriological and mycological cultures were performed on freshly collected surgical samples processed within 2–4 h, whereas FMDV RT-PCR/VP1 sequencing was performed on archived tissue aliquots for research purposes and did not influence treatment decisions.

This study’s limitations include the relatively small sample size and its restriction to buffaloes from a single governorate (Dakahlia). This retrospective design may have introduced inconsistencies and missing data (particularly regarding onset-to-presentation intervals) and precluded standardized assessment of systemic toxemia. In addition, the absence of contemporaneous control animals (buffaloes without tongue gangrene from the same region) and control feed samples from unaffected premises limits causal inference; therefore, the microbiological/mycological findings should be interpreted as associative rather than confirmatory. Furthermore, bacterial and fungal identification was presumptive based on culture characteristics and microscopy/Gram staining, without confirmation by biochemical tests, MALDI-TOF, or molecular methods, which may have limited species-level accuracy. Finally, only serotype O was detected in the examined tissues, despite using primers targeting multiple serotypes. Broader molecular characterization is needed to evaluate other serotypes across different epidemiological settings. Future studies should include broader geographic coverage, larger sample sizes, standardized systemic assessment, and appropriate controls.

## Conclusion

Overall, partial glossectomy performed along the demarcation line, combined with appropriate supportive therapy, resulted in satisfactory recovery in 93.2% of affected buffaloes. Prognosis was strongly influenced by the extent of devitalized tissue; extensive loss (≥ 12 cm) was associated with prolonged recovery, impaired feeding performance, and poor long-term outcome, leading to slaughter once systemic signs subsided. These findings highlight the clinical importance of careful assessment of tissue viability, timely removal of clearly devitalized tissue once diagnosed, along with aggressive supportive care to reduce systemic complications and restore feeding capacity.

## Data Availability

All data supporting the findings of this study are available within the paper.
